# Zinc/copper imbalance reflects immune dysfunction in human leishmaniasis: an ex vivo and in vitro study

**DOI:** 10.1186/1471-2334-4-50

**Published:** 2004-11-17

**Authors:** Johan Van Weyenbergh, Gisélia Santana, Argemiro D'Oliveira, Anibal F Santos, Carlos H Costa, Edgar M Carvalho, Aldina Barral, Manoel Barral-Netto

**Affiliations:** 1 LIMI, Gonçalo Moniz Research Center -Oswaldo Cruz Foundation (FIOCRUZ), Rua Waldemar Falcao 121, 40295-001 Salvador-BA, Brazil; 2 LIP, Gonçalo Moniz Research Center -Oswaldo Cruz Foundation (FIOCRUZ), Rua Waldemar Falcao 121, 40295-001 Salvador-BA, Brazil; 3 Institute for Immunological Investigation (iii, Instituto do Milênio), Sao Paulo-SP, Brazil; 4 Serviço de Imunologia, HUPES, Salvador-BA, Brazil; 5 Instituto de Quimica, UFBA, Salvador-BA, Brazil; 6 Infectious Disease Hospital, UFPI, Teresina-PI, Brazil

## Abstract

**Background:**

The process of elimination of intracellular pathogens, such as *Leishmania*, requires a Th1 type immune response, whereas a dominant Th2 response leads to exacerbated disease. Experimental human zinc deficiency decreases Th1 but not Th2 immune response. We investigated if zinc and copper levels differ in different clinical forms of leishmaniasis, and if these trace metals might be involved in the immune response towards the parasite.

**Methods:**

Blood was collected from 31 patients with either localized cutaneous (LCL), mucosal (ML) or visceral (VL) leishmaniasis, as well as from 25 controls from endemic and non-endemic areas. Anti-*Leishmania *humoral and cellular immune response were evaluated by quantifying specific plasma IgG, lymphoproliferation and cytokine production, respectively. Plasma levels of Cu and Zn were quantified by atomic absorption spectrophotometry.

**Results:**

A significant decrease in plasma Zn was observed in all three patient groups (p < 0.01 for LCL and ML, p < 0.001 for VL), as compared to controls, but only VL (7/10) and ML (1/7) patients displayed overt Zn deficiency. Plasma Cu was increased in LCL and VL (p < 0.001) but not in ML, and was strongly correlated to anti-*Leishmania *IgG (Spearman r = 0.65, p = 0.0028). Cu/Zn ratios were highest in patients with deficient cellular (VL<<LCL<ML) and exacerbated humoral (VL>LCL>ML) immune response. *Ex vivo *production of parasite-induced IFN-γ was negatively correlated to plasma Cu levels in LCL (r = -0.57, p = 0.01). *In vitro*, increased Cu levels inhibited IFN-γ production.

**Conclusions:**

1. Zn deficiency in VL and ML indicate possible therapeutic administration of Zn in these severe forms of leishmaniasis. 2. Plasma Cu positively correlates to humoral immune response across patient groups. 3. Environmentally or genetically determined increases in Cu levels might augment susceptibility to infection with intracellular pathogens, by causing a decrease in IFN-γ production.

## Background

Leishmaniasis is endemic in several parts of the world, with a global prevalence of over 12 million cases and 1.5–2 million new cases emerging every year [1]. The infection is caused by protozoan parasites of the genus *Leishmania*, transmitted through the bite of the sand fly vector. Several *Leishmania *species are able to cause a wide spectrum of clinical manifestations, ranging from the mild cutaneous form, the disfiguring mucosal form and the life-threatening visceral form, also known as kala-azar. In Brazil, *Leishmania (L.) braziliensis *causes cutaneous and mucosal disease, *L. amazonensis *causes cutaneous and, sporadically, visceral disease, while *L. chagasi *is exclusively associated with visceral disease. The clinical outcome of infection thus not only depends on the species involved, but also on the patient's immunocompetence. In recent years, a protective immune response against intracellular pathogens, such as *Leishmania, Listeria *and mycobacteria, has been defined as type 1 (Th1), whereas protection against extracellular pathogens, such as helminths, requires a type 2 (Th2) response. The murine model of experimental leishmaniasis has been instrumental for the elaboration of the Th1/Th2 paradigm, inasmuch as the preferential action of Th1 (IFN-γ, IL-12, TNF-α) or Th2 cytokines (IL-4, IL-5, IL-10) results in cure or progression of the disease, respectively [2,3]. In human leishmaniasis, this Th1/Th2 dichotomy is much less explicit for in vitro or ex vivo cytokine production. However, striking differences in cellular (lymphoproliferation and IFN-γ production) and humoral (total and anti-*Leishmania *IgG) immune response can be observed in different clinical forms of the disease. Our group has recently shown that patients with localized cutaneous leishmaniasis (LCL) display a diminished Th1 response during the early phase of disease, which is reverted after treatment [4]. In mucosal leishmaniasis (ML), on the other hand, an exacerbated Th1 response with increased IFN-γ and TNF-α levels, is believed to provoke tissue destruction [5]. In patients with visceral leishmaniasis (VL), characterized by immunosuppression and absence of IFN-γ production [6], we were able to show the beneficial effect IFN-γ *in vivo *[7]. Although zinc deficiency has been shown to lead to a selective Th1 deficiency in human volunteers [8], only few data are available on the role of trace elements in human leishmaniasis, being restricted to Old World LCL, showing increased serum copper and decreased serum zinc in Turkish LCL patients infected by *L. major *[9]. In this study, we investigated if Zn and Cu levels differ in different clinical forms of the disease, and if these trace metals might be correlated to anti-parasite immune response.

## Methods

Blood samples (10 ml heparinized tubes, Vacutainer) from 21 patients and 15 healthy controls (mostly patient's relatives) were obtained in an outpatient clinic in the district of Corte de Pedra, (Bahia state, Northeast of Brazil). This rural area has a low socio-economic status and a high incidence of infection with *Leishmania braziliensis *and, sporadically, *Leishmania amazonensis*. During a one year period, 14 patients with LCL (single lesion with less than 4 weeks of duration) were selected and treated (20 mg/kg of Sb (Glucantime) i.v. during 20 days). Of those, only 7 patients returned to draw blood after 3 months of treatment, but all patients cured during follow-up. Seven patients with ML were selected after several rounds of unsuccessful treatment and severe disease progression. Blood samples from 10 patients (at diagnosis, before treatment) with VL were obtained from two different urban areas (Salvador-Bahia and Teresina-Piaui). Ten healthy urban controls were recruited among students and laboratory staff (Salvador-Bahia). Diagnosis was confirmed by Montenegro skin test, serology, direct culture of parasites from lesions [4] or bone marrow aspiration for VL [7]. This study was approved by the Ethics Committee of the University Hospital Edgard Santos, Salvador.

Cu and Zn were quantified by atomic absorption spectrophotometry (Varian 220) using an air/acetylene flame. One ml of plasma was diluted tenfold with 0.05 % Triton X-100, 1 % HNO3, sonicated for 10 min and analyzed in triplicate. All reagents used were analytical grade (Merck). Due precautions were taken to avoid external and internal (hemolysis) trace metal contamination.

In order to investigate the influence of trace metals, antibodies and other endogenous plasma components, such as cytokines, on the *ex vivo *cellular immune response, we used a recently described model using whole blood and live *Leishmania *promastigotes [10], closely mimicking the early *in vivo *events following a sandfly bite. Whole blood was diluted tenfold in culture medium (RPMI supplemented with L-glutamine and gentamycin, all from Gibco-BRL) and stimulated with *L. amazonensis *promastigotes (10^5^/mL). Buffy coats from normal blood donors were used to obtain large quantities of cells required for *in vitro *experiments to examine the effect of exogenous trace metals upon cytokine production. Mononuclear cells were separated by density gradient centrifugation (Ficoll-Paque, Pharmacia, Uppsala, Sweden) and cultured in complete culture medium (supplemented with 10 % fetal calf serum, Gibco-BRL). Cu and Zn concentrations were below the detection limit in RPMI and less than 2 μM in complete culture medium. Supernatants were collected after 72 h of culture and frozen in aliquots for cytokine determination. Lymphoproliferation was quantified by measuring [H]-thymidine incorporation after 120 h of culture. IFN-γ, TGF-β1, TNF-α and IL-5 in plasma or culture supernatants were quantified using commercial ELISA kits (DuoSet, R&Dsystems). All results are expressed as mean ± SEM. Statistical evaluation of data was performed using GraphPad Prism software: Mann-Whitney test for comparing patients and control groups, Spearman rank test for correlation and Wilcoxon signed rank test for comparing *in vitro *treatments; a p-value <0.05 was considered significant.

## Results

**Figure 1:**
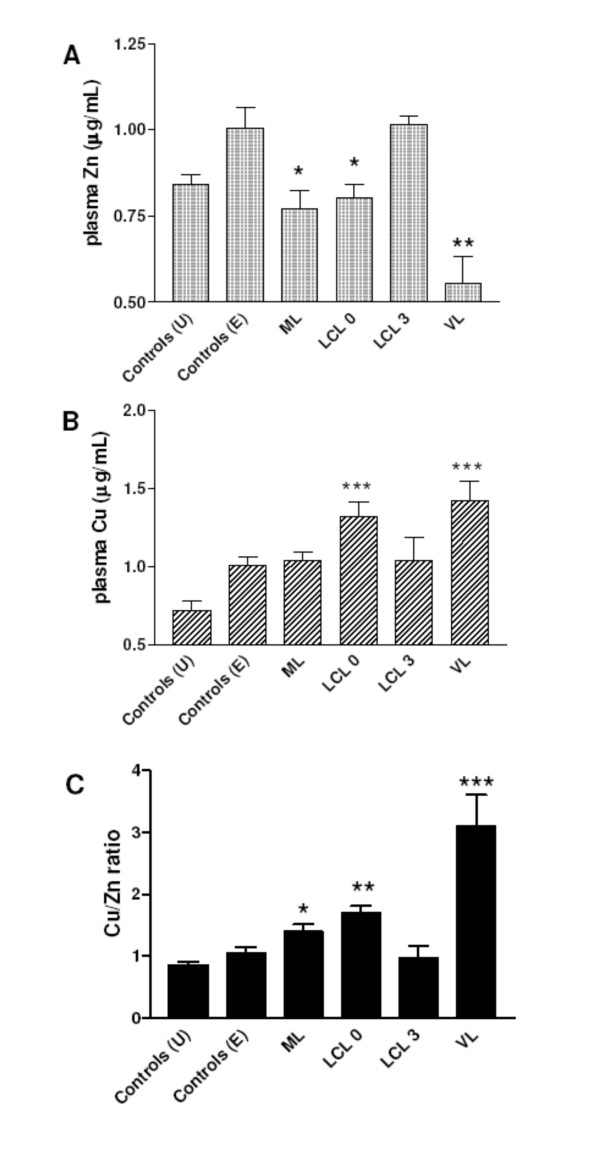
**Plasma levels of Zn and Cu in controls and patients**. Bars represent mean (+/-SEM) Zn (**A**) and Cu (**B**) plasma levels, and plasma Cu/Zn ratios (**C**) of urban (U) and endemic (E) controls; and patients with mucosal leishmaniasis (ML), localized cutaneous leishmaniasis before (LCL0) and after 3 months of treatment (LCL3), and visceral leishmaniasis (VL). *p < 0.05, **p < 0.01, ***p < .001 for LCL0 and ML *vs*. E and for VL *vs*. U (Mann-Whitney test).

A significant decrease in plasma Zn was observed for both LCL and ML patients, as compared to controls from the same endemic area (0.80 ± 0.04 and 0.77 ± 0.05 vs. 1.01 ± 0.06 μg/mL, p < 0.01, Figure 1A), and in VL patients, as compared to urban controls (0.55 ± 0.08 vs. 0.83+/-0.03 μg/mL, p < 0.001, Figure 1A). Zn deficiency (plasma Zn <0.65 μg/mL), however, was observed only in VL (7/10) and ML (1/7) patients. As shown in Fig. 1B, plasma Cu was significantly increased in LCL (1.32 ± 0.10 vs. 1.01 ± 0.05 μg/mL, p < 0.001) and VL (1.42 ± 0.13 vs. 0.72 ± 0.06 μg/mL, p < 0.001), but not in ML (1.04 ± 0.05 vs. 1.01 ± 0.05 μg/ml). After three months of treatment, plasma Zn increased and Cu decreased in LCL patients, resulting in values indistinguishable from endemic controls. Although within normal physiological ranges [11], Cu and Zn levels were significantly increased in healthy controls from the endemic area, as compared to urban controls (p < 0.01, Fig, 1A and 1B). Cu/Zn ratios, however, were similar in both control groups (p = 0.12), but significantly increased in all three patient groups (Fig. 1C), reaching a three-fold molar excess of Cu to Zn in VL patients.

**Figure 2:**
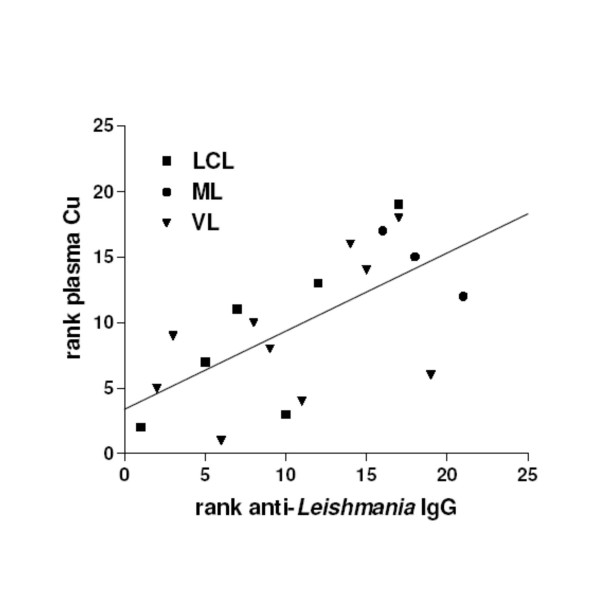
**Correlation between plasma Cu and anti-*Leishmania *IgG**. Plasma levels of Cu and anti-*Leishmania *IgG were positively correlated in patients with localized cutaneous leishmaniasis before treatment (LCL), mucosal leishmaniasis (ML) and visceral leishmaniasis (VL) (Spearman r = 0.65, p = 0.0028).

**Figure 3:**
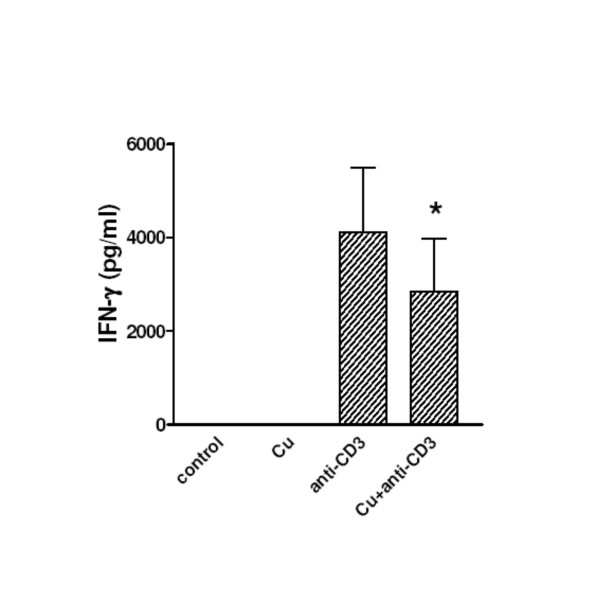
**In vitro effects of exogenous Cu upon IFN-γ production in healthy controls**. Mononuclear cells from normal donors were incubated for 48 h in the absence or presence of anti-CD3 (200 ng/ml), Cu (10 μM CuCl^2^) or both. Bars represent mean (+/-SEM) IFN-γ concentration in supernatants from six different donors. Unstimulated cells or cells stimulated with Cu alone did not produce IFN-γ. *p < 0.05 *vs*. anti-CD3-stimulated cells (Wilcoxon signed rank test).

To determine if these observations reflect possible changes in the patients' immune status, we quantified humoral and cellular anti-*Leishmania *immune response *ex vivo *and correlated them to trace element levels. When comparing patient groups, a stepwise increase in anti-*Leishmania *IgG and Cu/Zn ratio can be observed, with ML<LCL<VL (Table I and Fig. 1). In addition, a highly significant correlation between plasma Cu, but not Zn or Cu/Zn ratio, and anti-*Leishmania *IgG was observed across patient groups (Fig. 2, Spearman r = 0.65, p = 0.0028). Since lymphoproliferation and IFN-γ production were virtually absent in VL, correlation with trace element levels across patient groups could not be calculated, but an inverse order was observed, with ML>LCL>>VL (Table I) as compared to Cu/Zn ratio with ML<LCL<<VL (Fig. 1), confirming reciprocal regulation of Th1/cellular immune response and Th2/humoral immune response. However, we found a significant negative correlation between plasma Cu and *ex vivo *IFN-γ production (Spearman r = -0.86, p = 0.024) in untreated patients only, whereas no significant correlation was observed for *Leishmania*-or mitogen-induced lymphoproliferation, TGF-β1, TNF-α and IL-5 levels in plasma or culture supernatants (not shown). To verify the hypothesis that increased Cu levels might down-regulate IFN-γ production, we added Cu (10 μM, corresponding to the increase of plasma Cu observed ex vivo in LCL patients) to mitogen-or anti-CD3-stimulated *in vitro *cultures from healthy controls. As shown in Figure 3, 10 μM of CuCl_2 _significantly decreased anti-CD3-induced IFN-γ production (40.5 ± 9.3 % inhibition, p < 0.05). Interestingly, the addition of physiological concentrations of Zn (10–30 μM) to *ex vivo *or *in vitro *cultures did not revert the apparently inhibitory effect of endogenous or exogenous Cu on IFN-γ secretion. (not shown).

## Discussion

Although plasma Zn was significantly decreased in all three patient groups, plain Zn deficiency was only observed in seven VL patients and one ML patient, being absent in LCL patient and in both control groups. In parallel, plasma Cu in VL patients increased to levels which have been shown to be toxic *in vitro *[12]. In addition, a highly significant positive correlation between plasma Cu and parasite-specific IgG across patient groups suggests that the trace element might interfere in anti-*Leishmania *immune response, e.g. by leading to a non-protective Th2/humoral immune response, known to be exacerbated in visceral leishmaniasis. Increased plasma Cu cannot be considered as a mere marker of inflammation, since it was not observed in ML, a chronic inflammatory condition characterized by high production of pro-inflammatory cytokines, such as TNF-α and IFN-γ [5]. Absence of correlation between TNF-α and trace metal levels underscores the specificity of the inhibitory effect of Cu upon IFN-γ production, which might in fact be the upstream event to an increased humoral anti-*Leishmania *response.

Since our *in vitro *findings indicate Zn as a monocyte/macrophage activator [13], the significant decrease in Zn in ML patients might be responsible for the inability of the patients to clear the parasite, in spite of high IFN-γ levels and several rounds of treatment. LCL patients represent an intermediate group between ML and VL, displaying a detectable, but variable humoral and cellular immune response with production of both Th1 and Th2 cytokines, undergoing a shift towards the Th1/cellular pole after successful treatment [4]. We observed a reciprocal association between Cu/Zn levels and humoral and cellular immune response between the three patient groups (Table I and Fig. 1), as well as a complete reversal of increased Cu/Zn ratios after treatment in LCL patients. Taken together, these data indicate that Cu/Zn imbalance might serve as a marker for decreased Th1 response and immunodeficiency in leishmaniasis, being more pronounced in its most severe and possibly fatal visceral form. Unfortunately, no clinical follow-up was possible in VL patients, but mortality, mostly due to co-infections, and therapeutic failure occur in 6,1 % of the cases [14,15].

It is tempting to speculate that environmental exposure to copper might increase susceptibility to *Leishmania *and other intracellular pathogens, such as *Listeria *and mycobacteria, e.g. by directly interfering with cytokine production as previously shown [16]. Increased plasma Cu in endemic controls might reflect the use of Cu as a fungicide in cocoa plantations in the Corte de Pedra area. It should be stated that spouses and relatives were preferentially chosen as endemic controls, since environmental exposure and nutritional status are far more important determinants for Cu and Zn levels than sex or age [11]. In addition, (epi)genetic factors related to copper homeostasis might render normal individuals more susceptible to copper toxicity [17]. Thus, increased Cu levels and decreased Zn levels might be a cause, rather than a consequence of LCL. On the other hand, absence of Cu increase, linked to uncontrolled IFN-γ production, might underlie evolution towards ML. Long-term follow-up of treated patients and comparison with other endemic areas might learn if trace metal levels have predictive value for clinical evolution of and/or susceptibility to leishmaniasis. In addition, we propose that trace metal levels should be taken into account in vaccine strategies for leishmaniasis, because of the importance of Zn in a protective Th1 response [8] and because of the possibly deleterious effect of Cu described in this study. Two recent large-scale vaccination trials for cutaneous and visceral leishmaniasis [18,19] were carried out in regions where Zn deficiency is prevalent, namely Iran and Sudan, which might have contributed in part to the low protection rate observed in both trials.

Administration of Zn in vivo has been shown to down-regulate increased Cu levels in patients with Wilson disease and to revert its toxicity [20], suggesting that systemic administration of Zn might be beneficial in addition to its direct immunostimulatory effect [8]. A recent report [21] demonstrated the safety and efficiency of oral Zn in Old World cutaneous leishmaniasis, a mild and self-healing form of the disease, with patients displaying normal Zn levels. Our results strongly suggest that zinc therapy should be considered in the mucosal and visceral forms of leishmaniasis, associated with high morbidity and mortality, as well as frequent failure of antimonial therapy.

## Conclusions

Zn deficiency in visceral and mucosal leishmaniasis indicate possible therapeutic administration of Zn in these severe forms of leishmaniasis. Plasma Cu positively correlates to (non-protective) humoral immune response across patient groups, and reciprocally, increased Cu levels decreased *in vitro *(protective) IFN-γ production, implying that environmentally or genetically determined increases in Cu levels might augment susceptibility to infection with intracellular pathogens. Our data indicate that Cu/Zn imbalance can be a useful marker for immune dysfunction in leishmaniasis and suggest that trace metals are implicated in both humoral and cellular anti-*Leishmania *immune response, which should inspire future strategies for therapy and immunoprophylaxis of human leishmaniasis.

## Competing interests

The author(s) declare that they have no competing interests.

## Authors' contributions

JVW designed the scientific project, analyzed the data and wrote the paper. GS was responsible for sample processing and data collection. AD selected LCL and ML patients, as well as controls from the endemic area. CHC selected VL patients. AFS did the trace metal analysis. AB and EMC supervised the field work and reviewed the paper. MBN analyzed the data and reviewed the paper.

## Pre-publication history

The pre-publication history for this paper can be accessed here:



## Figures and Tables

**Table I:** Characteristics and immune parameters of patient groups and controls

**Group (n)**	**Age**	**Male/Female**	**Anti-*Leishmania *IgG (O.D.)**	**Lympho-proliferation (cpm)^b^**	**IFN-γ (pg/ml)^b^**
**Urban controls (10)**	24.9 (2.0)	7/3	< cut-off	< 100	< 20
**Endemic controls (15)**	28.7 (8.8)	3/12	< cut-off^a^	ND	< 20
**LCL0 (14)**	28.4 (12.7)	10/4	0.178 (0.073)	2737 (1178)	612 (207)
**LCL3 (7)**	28.5 (11.2)	5/2	0.074 (0.021)	ND	685 (348)
**ML (7)**	44.4 (11.9)	5/2	0.016 (0.005)	> 5000	> 2000
**VL (10)**	18.4 (3.8)	6/4	0.188 (0.072)	< 100	< 20

Results are expressed as mean (SEM). ND: not done. LCL0 and LCL3, localized cutaneous leishmaniasis at 0 and 3 months of treatment, ML mucosal leishmaniasis, VL visceral leishmaniasis.

^a ^One endemic control displayed positive serology and was discarded from analysis.

^b ^Following stimulation of tenfold diluted whole blood with live *Leishmania *promastigotes.
